# Dual Oxidase, a Hydrogen-Peroxide-Producing Enzyme, Regulates Neuronal Oxidative Damage and Animal Lifespan in *Drosophila melanogaster*

**DOI:** 10.3390/cells11132059

**Published:** 2022-06-29

**Authors:** Minwoo Baek, Wijeong Jang, Changsoo Kim

**Affiliations:** School of Biological Sciences and Technology, Chonnam National University, Gwangju 61186, Korea; minwoo.back@gmail.com (M.B.); kimoe@naver.com (W.J.)

**Keywords:** lifespan, duox, ROS, *Drosophila melanogaster*

## Abstract

Reducing the oxidative stress in neurons extends lifespan in *Drosophila melanogaster*, highlighting the crucial role of neuronal oxidative damage in lifespan determination. However, the source of the reactive oxygen species (ROS) that provoke oxidative stress in neurons is not clearly defined. Here, we identify *dual oxidase* (*duox*), a calcium-activated ROS-producing enzyme, as a lifespan determinant. Due to the lethality of *duox* homozygous mutants, we employed a *duox* heterozygote that exhibited normal appearance and movement. We found that *duox* heterozygous male flies, which were isogenized with control flies, demonstrated extended lifespan. Neuronal knockdown experiments further suggested that *duox* is crucial to oxidative stress in neurons. Our findings suggest *duox* to be a source of neuronal oxidative stress associated with animal lifespan.

## 1. Introduction

Hydrogen peroxide (H_2_O_2_) is a reactive oxygen species (ROS) that functions in signaling pathways by specifically oxidizing redox-sensitive proteins in diverse biological processes [1,2,3,4]. However, the upregulated or dysregulated production of H_2_O_2_ in combination with diminished anti-ROS activity confers oxidative stress that abrogates redox-dependent signaling or nonspecifically and irreversibly damages macromolecules, in turn accelerating the aging process and decreasing lifespan [5,6,7,8,9]. Neurons are particularly susceptible to oxidative stress, and the elevation of the antioxidant power in neurons has been shown to extend lifespan [10,11,12,13,14]. Various anti-ROS enzymes and anti-ROS transcriptional networks are well known, yet the source of ROS production that provokes oxidative stress in neurons is not yet completely understood.

ROS production is inducible by members of the NADPH oxidase (NOX) family; such induced production is distinct from the steady-state ROS production that occurs through oxidative respiration in mitochondria [15]. Dual oxidase (Duox) is a member of the Nox family, with an EF-hand motif that is activated by intracellular calcium to produce H_2_O_2_ [1,16,17]. It is widely expressed in the nervous system in rats, zebrafish, and *Drosophila* [18,19,20,21]. Inflammation, wounds, and various signals that increase intracellular calcium ion concentration all activate *duox* [22,23,24,25] and, in particular, UV irradiation, ROS, and p38 MAPK signaling increase its expression [18,26,27,28,29,30]. *Duox* is known to mediate pain hypersensitivity due to UV-induced damage [31] and, in the CNS, axon regeneration after wounds [32,33]. However, the full role of *duox* in the nervous system still remains largely unknown. In *Drosophila*, there is only a single *duox* gene, in contrast to the two genes present in mammals [21]. Here, we show that through its generation of H_2_O_2_, *duox* contributes to oxidative stress in neurons and is a determinant of *Drosophila melanogaster* lifespan.

## 2. Materials and Methods

### 2.1. Fly Strains and Maintenance

*Tub-Gal4, elav-Gal4, Mef-Gal4, UAS-duox^RNAi^* (#38907 and #32903), and *duox^KG07745^* were purchased from the Bloomington Drosophila Stock Center. *UAS-duox^RNAi^* (#38907 and #32903) and *duox^KG07745^* were outcrossed with *w^1118^* for ten generations to eliminate background effects. Flies were raised on standard cornmeal fly food at 25 °C and 50% humidity in a 12-hour light/dark-cycle incubator.

### 2.2. Lifespan and Oxidative Stress Assays

For lifespan assays, 20 flies per vial were transferred to fresh food vials every two days. Dead flies were counted every day. For survival under oxidative stress, 5-day-old flies were starved for 6 h at 25 °C in vials containing water-soaked tissues and then transferred to vials containing either normal food with 5 mM methyl viologen hydrate (paraquat) or 5% sucrose-agar medium with 5% hydrogen peroxide [34]. Dead flies were counted every 12 h.

### 2.3. Locomotive Activity

Negative geotactic movement assays were performed as described previously [35]. Briefly, a vial (9.5 cm × 2 cm) containing 10 flies was tapped to send the flies to the bottom. Flies that crossed the 8-centimeter line from the bottom within 10 s were scored. In total, 100 male flies were assessed in 10 vials (10 flies per vial).

### 2.4. RT-PCR and Real-Time PCR

Total RNA was extracted from 20 flies using Trizol reagent (Invitrogen, Grand Island, NY, USA) and cDNA was obtained by reverse transcription (TOPscript RT Mix; Enzynomics, Seoul, Korea). Real-time PCR was conducted using SYBR green PCR Master Mix on the ABI PRISM 7500 system (Applied Biosystem, Foster City, CA, USA). The primer pairs used were as follows. *Duox*: CAGACCGAGAAACAGCGCTAC, AAACAGCCGGCTGAGCCTGCG; *rp49*: CACCAGGAACTTCTTGAATCCGG, AGATCGTGAAGAAGCGCACC; *catalase*: CGGCTTCCAATCAGTTGAT, GATGTGAACTTCCTGGATGAG; *dSOD1*:CAAGGGCACGGTTTTCTTC, TCCGGACCGCACTTCAATC; *dSOD2*: AATTTCGCAAACTGCAAGC, ACCACCAAGCTGATTCAGC.

### 2.5. Determination of Total ROS Levels

Total ROS was measured as previously described [36]. Briefly, 10 flies or the heads of 50 flies were homogenized in 200 μL of 50 mM Tris-HCl, pH 7.4, on ice. Extracts were centrifuged at 13,200 rpm for 10 min at 4 °C. Homogenates were incubated with H_2_DCFDA (Life Technologies, Carlsbad, CA, USA) at 5 μM in a 200-microliter reaction volume at 37 °C for 15 min in darkness, carried out on 96-well plates. Florescence intensity was monitored on a Gemini XPS fluorescence microplate reader (Molecular Devices, Sunnyvale, CA, USA) set for 488 nm excitation and 520 nm emission. Florescence intensity was normalized by protein amount measured by Bradford method [37]. All experiments were carried out with three biological replicates.

### 2.6. Determination of H_2_O_2_ Levels

H_2_O_2_ was measured as previously described [36]. Briefly, 10 flies were homogenized in phosphate saline buffer (50 mM, pH 7.4) containing aminotriazol (2 mg/mL) and incubated at 4 °C for 15 min. The extracts were centrifuged at 13,200 rpm for 10 min at 4 °C to obtain supernatants. H_2_O_2_ levels were measured using a hydrogen peroxide assay kit (Cayman Chemical, Ann Arbor, MI, USA) on a microplate reader (VersaMax; Molecular Devices) set to 590 nm. H_2_O_2_ levels were normalized according to protein amount measured by Bradford method [37]. All experiments were carried out with three biological replicates.

### 2.7. Determination of Protein Oxidation

Carbonyl groups in proteins were detected using an oxyblot protein oxidation detection kit (Milipore, Billerica, MA, USA) according to the manufacturer’s instructions. Briefly, proteins were extracted from 10 flies or ~50 fly heads using RIPA buffer, reacted with 2,4-dinitophenylhydrazine (5 mM) at 25 °C for 100 min, and then subjected to SDS/PAGE and Western blotting using an antibody to the dinitrophenyl moiety. The blots were developed with ECL (Amersham, Buckinghamshire, UK) and the images were captured with a Vilber Lourmat Fusion FX (Vilber Lourmat, Eberhardzell, Baden-Württemberg, Germany). Band intensities were determined with the Fusion program (Vilber Lourmat). Finally, blots were stripped with a stripping buffer (Thermo Fisher scientific, Waltham, MA, USA), and then by a separate Western blot carried out using β-actin antibody (Santa Cruz, Dallas, TX, USA), which was used as loading control. All experiments were carried out with three biological replicates.

### 2.8. Determination of DNA Oxidation

The level of 8-hydroxydeomyguanosine (8-OHdG) was measured using the OxiSelect^TM^ Oxidative DNA Damage ELISA kit (Cell Biolabs, San Diego, CA, USA). Briefly, genomic DNA from 10 flies or ~50 fly heads was extracted using PureLink^TM^ genomic DNA kits (Invitrogen, Carlsbad, CA, USA), treated at 95 °C for 5 min, and digested with nuclease P1 (20 mM sodium acetate, pH 5.2) and alkaline phosphatase (100 mM Tris, pH 7.5). Samples were centrifuged for 5 min at 6000× *g* and the supernatants were used for ELISA assays following the manufacturer’s instructions. The amount of 8-OHdG in each sample was calculated based on an 8-OHdG standard curve.

### 2.9. TUNEL Labeling

Adult fly brains were dissected in M3 medium (Merck, Boston, MA, USA), fixed in 4% paraformaldehyde in phosphate buffered saline (PBS, 50 mM, pH 7.4) for 20 min at room temperature, washed with PBS, and permeabilized by 2 min incubation in PBS containing Triton X-100 (0.1%) on ice. After washing with PBS three times, the samples were first incubated in Na citrate (0.1 M, pH 6.0) for 10 min at 65 °C, followed by incubation in Tris-HCl buffer (0.1 M, pH 7.5) containing 3% BSA and 20% bovine serum albumin for 30 min. After washing three times with PBS, the samples were incubated in TUNEL assay solution (In Situ Cell Death Detection Kit; Roche, Basel, Switzerland) for 1 h at 37 °C, in accordance with the manufacturer’s recommendations. After washing three times with PBS, the samples were mounted in mounting solution (Prolong Gold Antifade reagent; Life Technologies) and examined by confocal microscopy (Leica TCS SP5; Leica Microsystems, Morrisville, NC, USA).

### 2.10. Statistical Analysis

The statistical significance of differences in standard error of the mean (SEM) values was obtained using Student’s *t*-test (two-tailed), analysis of variance (ANOVA) with post hoc analysis (Dunnett’s tests, two-tailed). Kaplan-Meier survival curves were used for lifespan and survival analysis with log-rank tests. Prism software (GraphPad version 6.0, San Diego, CA, USA) was used to calculate *p*-values. Results were considered statistically significant at levels * *p* < 0.05, ** *p* < 0.01, *** *p* < 0.001, and **** *p* < 0.0001.

## 3. Results

**Figure 1:**
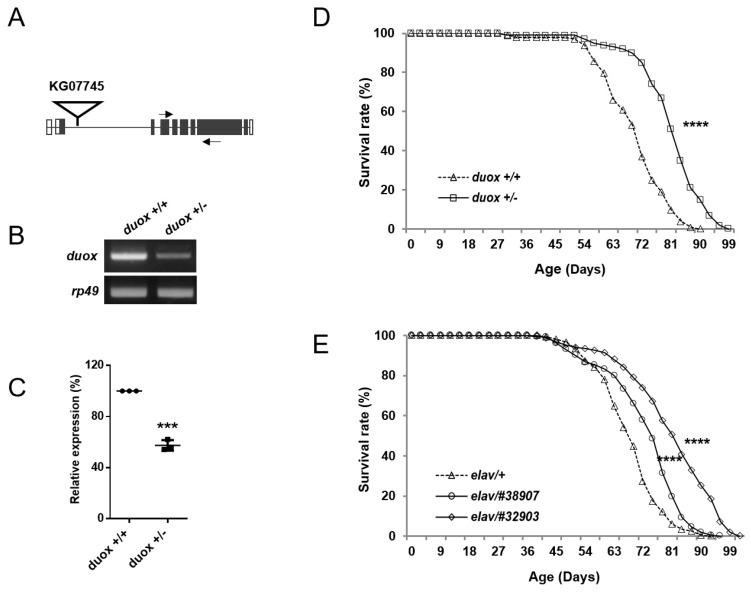
Extension of male lifespan with *duox* heterozygosity and neuronal *duox* knockdown. (**A**) Diagram showing the location of the P-element (KG07745), which is inserted in the second intron of *duox*. Empty and filled squares denote noncoding and coding regions of exons, respectively. Arrows denote primers used for RT-PCR. (**B**) Agarose-gel image of RT-PCR products following amplification of the *duox* gene from control (*w^1118^*, denoted *duox*^+/+^) and heterozygous (*duox ^KG07745/+^*, denoted *duox*^+/−^) flies. *rp49* was used as a loading control. Three independent experiments carried out with similar results. (**C**) qRT-PCR of *duox* gene, with *rp-49* used as a control. Relative expression denotes *duox* transcript level. Averages and standard errors were derived from three independent experiments. Student’s *t*-test, *** *p* < 0.001. (**D**) Lifespan of wild-type and *duox* heterozygous male flies. Twenty flies/vial, total twenty vials. Log-rank test, **** *p*< 0.0001. *duox*^+/+^ denotes *w^1118^* flies. *duox*^+/−^ denotes *duox^+/kG07745^* flies, which were isogenized ten times with control flies (*w^1118^*). (**E**) Lifespans of *elav-Gal4 > UAS-duox^RNAi^* flies. *elav/#38907* and *elav/#32903* denote *elav-Gal4 > UAS-duox^RNAi^* #38907, *elav-Gal4/+*; *UAS-duox^RNAi^* #32903/+. Twenty flies/vial, total ten vials, male flies. Log-rank test, **** *p* < 0.0001. *UAS-duox^RNAi^* (*#38907, 32903*) flies were isogenized with control *w^1118^* flies ten times to reduce genetic background differences.

To find genes involved in lifespan extension, we carried out a small-scale screening of mutants with extended lifespan, which identified *duox* heterozygous (*duox^KG07745/+^*) male flies of interest. In the transgenic allele, the P-element KG07745 was inserted into the second intron of the *duox* locus, which reduced the *duox* transcripts to ~half the level in control flies (Figure 1A–C). As the insertion was downstream of the initiation codon, the Duox protein structure was disrupted, suggesting that the *duox^KG07745^* allele was probably null or severely hypomorphic with regards to protein function The. *Duox^KG07745^* homozygotes were lethal, while the *duox^KG07745^* heterozygotes were normal in both appearance and movement (Figure S1).

Initial screening involved a small number (*n* = 40) of unisogenized *duox* heterozygous males. To ensure rigor, we isogenized the *duox* heterozygous flies with the control flies (*w^1118^*) ten times to reduce genetic background differences, and increased the population to 400 flies, with 20 flies per vial given fresh food every two days. We focused on the *duox* heterozygous male flies due to the small effect of the allele on the lifespans of the heterozygous female flies (Figure S2A). The control male flies (*w^1118^*) lived for a median of 70 days; meanwhile, the isogenized *duox* heterozygous males had an extended median lifespan of 81 days, which was an increase of 15% (Figure 1D, Table S1). Next, we employed the bipartite *Gal4/UAS* expression system to knock down the *duox* function across the whole body, including in both muscle and neurons. Rigorously, we isogenized the *UAS-duox^RNAi^* (*#38907, 32903*) flies with *w^1118^* flies ten times to reduce the genetic background differences between the *Gal4 > UAS-duox^RNAi^* flies and the control *Gal4 > w^1118^* flies. Driving the whole-body *duox^RNAi^* (#38907 and #32903) expression with the whole-body *Gal4* (*tub-Gal4*) was lethal. A second experiment using muscle-only *Gal4* (*mef-Gal4*) did not demonstrate pronounced lifespan extension in the males or females (Figure S2C,D). However, driving the *duox* RNAi with the pan-neuronal *Gal4* (*elav-Gal4*) extended the male lifespan by 10% and 20%, respectively, for the *duox^RNAi^* #38907 and #32903 relative to the *elav-Gal4/+* control (Figure 1E, Table S1); the lifespan extension in the females was not pronounced (Figure S2B).

**Figure 2:**
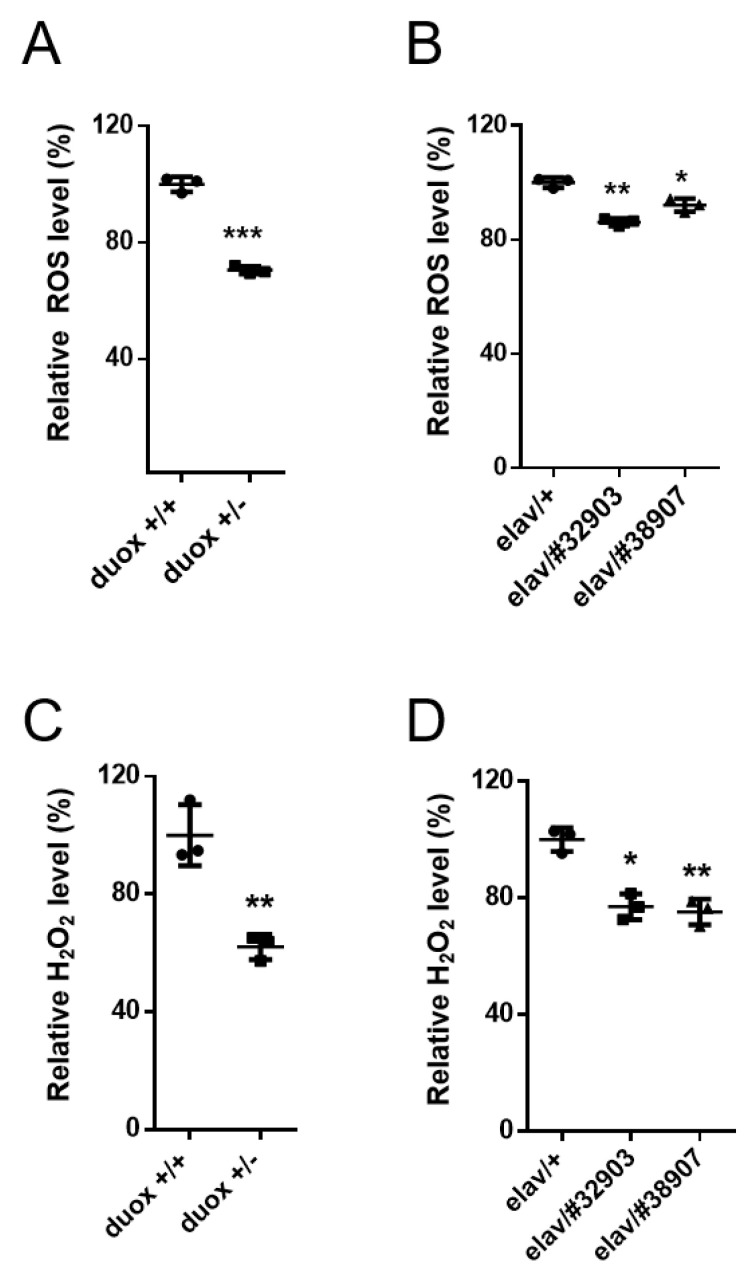
Decrease in global ROS and H_2_O_2_ levels with *duox* heterozygosity and neuronal *duox* knockdown. Global ROS (**A**,**B**) and H_2_O_2_ (**C**,**D**) levels normalized to protein concentration were derived for whole-fly extracts (**A**,**C**) and head extracts (**B**,**D**). Values for *duox*^+/+^ (**A**,**C**) and *elav*/+ (**B**,**D**) were set to 100%. Error bars represent SEM of three replicates using 5-day-old male flies. Student’s *t*-test, * *p* < 0.05, ** *p* < 0.01, *** *p* < 0.001. *duox*^+/+^ and *duox*^+/−^ denote *w^1118^* and *duox^+/kG07745^*. *elav*/#*38907, 32903* denotes *elav-Gal4*/*UAS-duox^RNAi^ #38907, elav-Gal4/+*; *UAS-duox^RNAi^* #32903/+.

**Figure 3:**
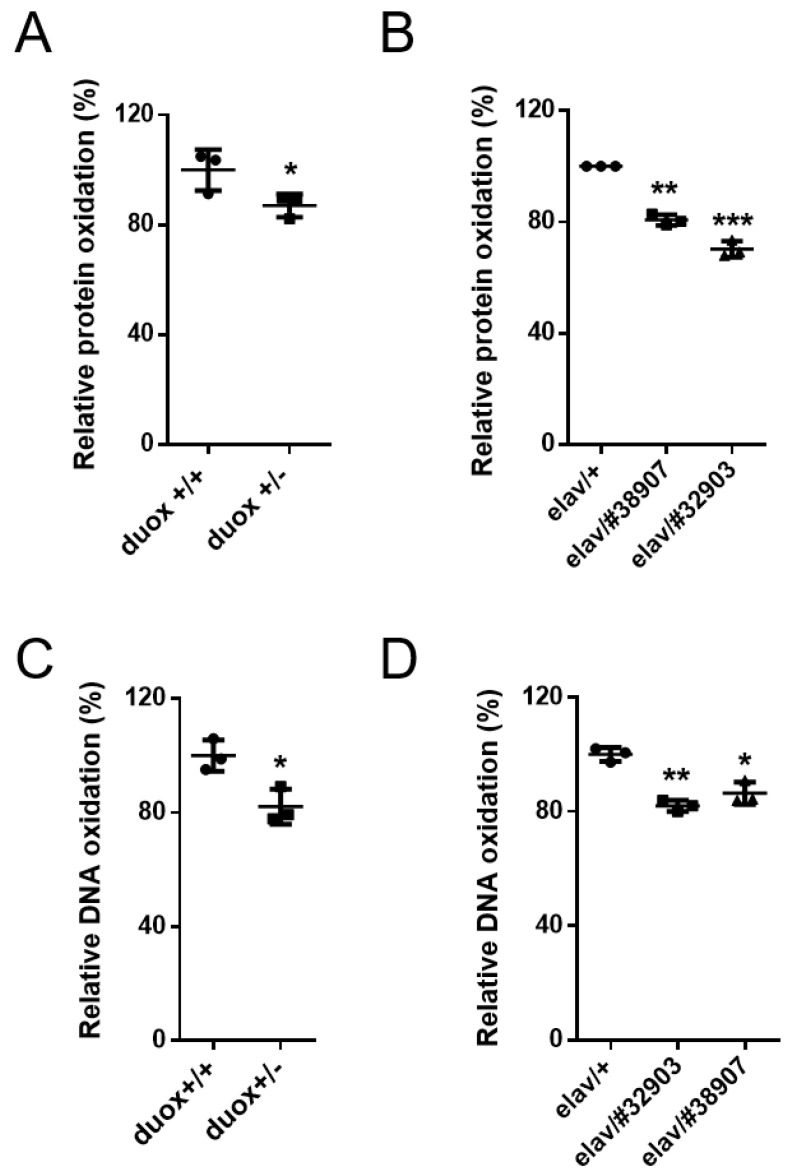
Decrease in oxidative damage with *duox* heterozygosity and neuronal *duox* knockdown. Relative protein and DNA oxidation levels in whole-fly extracts (**A**,**C**) and in head extracts (**B**,**D**). Data were set to *duox*^+/+^ (**A**,**C**) and elav/+ (**B**,**D**) at 100%. Error bars represent SEM of three replicates using 60-day-old (**A**) and 45-day-old male flies (**B**–**D**). Three replicates. Error bars represent SEM. Student’s *t*-test, * *p* < 0.05, ** *p* < 0.01, and *** *p* < 0.001. *duox*^+/+^ and *duox*^+/−^ denote *w^1118^* and *duox^+/kG07745^*, respectively. *elav*/#*38907, 32903* denotes *elav-Gal4*/ *UAS-duox^RNAi^ #38907, elav-Gal4/+*; *UAS-duox^RNAi^* #32903/+.

As Duox is a ROS-producing enzyme, the *duox* heterozygotes (*duox*^+/−^) would be expected to produce less ROS than the control flies (*duox*^+/+^). The global ROS was measured using the non-fluorescent compound 2′,7′-dichlorodihydrofluorescein diacetate (DCFH-DA), which reacted with the ROS to produce fluorescent dichlorofluorescein (DCF). The ROS-dependent DCF fluorescence intensity was normalized to the protein concentration in each extract. The whole-fly extracts from the *duox* heterozygotes produced less ROS than those from the controls (Figure 2A). The head extracts from the flies with the neuronal knockdown of the *duox* also exhibited less ROS than the controls (Figure 2B). Similarly, the assays of the H_2_O_2_ production revealed that the *duox* heterozygote whole-fly extracts produced less H_2_O_2_ than those from the controls (Figure 2C). The same was true of the head extracts from the flies with the neuronal knockdown of the *duox* (Figure 2D). ROS mediates the oxidation of amino acid residues in proteins and of the bases in DNA [38,39,40], and this oxidation was decreased in the whole-body extracts of the *duox* heterozygous male flies (Figure 3A,C). Similarly, the neuronal knockdown of the *duox* via RNAi resulted in reduced protein and DNA oxidation in the head extracts (Figure 3B,D).

**Figure 4:**
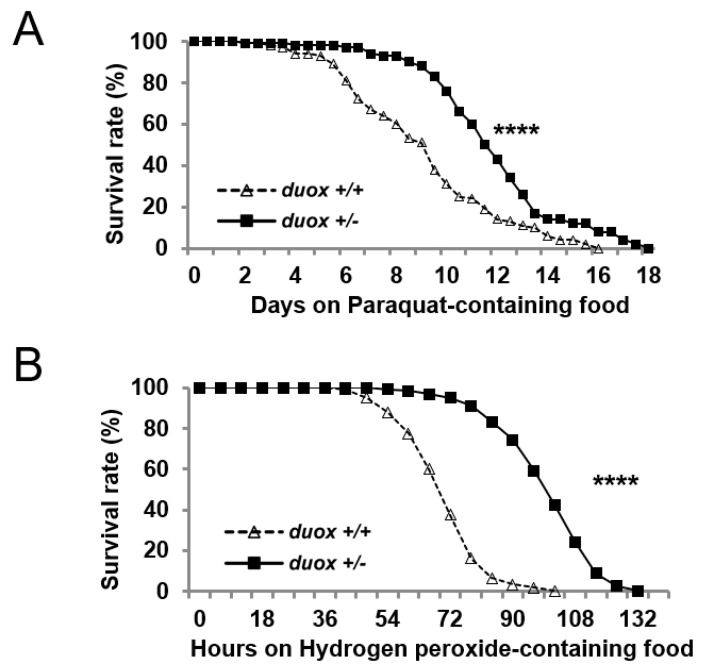
ROS resistance associated with *duox* heterozygosity. Survival of 5-day-old male flies on food containing 10 mM paraquat (**A**) and 10% hydrogen peroxide (H_2_O_2_) (**B**). *n* = 200 (20 flies/vial, total 10 vials). Log-rank test, **** *p* < 0.0001. *duox*^+/+^ denotes *w^1118^* flies. *duox*^+/−^ denotes *duox^+/kG07745^* flies, which were isogenized ten times with control flies (*w^1118^*).

Given that the *duox* heterozygous males experienced less oxidative damage, next, we examined whether these males exhibited increased survival when provided with ROS-producing food. When given food-containing paraquat, an agent that produces cellular ROS, the *duox^KG07745^* heterozygous males exhibited increased survival; they also showed increased survival on H_2_O_2_-containing food (Figure 4A,B). Oxidative stress is relevant to apoptotic cell death and neurodegeneration, suggesting that apoptotic cells might be reduced by *duox* reduction. Indeed, the apoptotic cells in the brain were reduced by the *duox* heterozygosity and by its neuronal knockdown (Figure S3). The observed ROS decrease could have been due to increased levels of anti-ROS enzymes. However, the levels of the anti-ROS enzyme catalase, dSOD1, and dSOD2 were constant with age regardless of the *duox* status (Figure S4), suggesting that the lower ROS level in the *duox* heterozygous flies was not due to anti-ROS enzymes, but to *duox* heterozygosity.

## 4. Discussion

Neurons are vulnerable to oxidative stress, which is related to neuronal aging, neuropathies, and lifespan. Considerable attention has been paid to antioxidant defense systems, but the ROS sources that oxidize macromolecules in neurons are not yet well defined. Here, we identify Duox, a calcium-activated NADPH oxidase, as a determinant of neuronal oxidative stress and lifespan. We found that neuronal oxidative damage was reduced and lifespan was extended by the neuronal knockdown of *duox* in *Drosophila melanogaster*. Our findings suggest that *duox* is a source of ROS production in neurons, which affects lifespan.

It is intriguing that oxidative stress, particularly stress in neurons, should impact lifespan. It was previously shown that lifespan can be extended by enhancing anti-oxidative power in neurons, specifically through the neuronal overexpression of Peroxiredoxin 4 (*prx4*), an anti-ROS protein, and of glutamate-cysteine ligase, an enzyme that catalyzes the biosynthesis of the reducing agent, glutathione [13,41]. Similarly, the neuronal knockdown of Kelch-like ECH-associated protein 1 (*keap1*) increases longevity [8,12]; this gene encodes an inhibitor of the cap ’n’ collar (CncC) transcription factor, the *Drosophila* homolog of the mammalian nuclear factor E2-related factor 2 (NRF2), which provides a major anti-ROS cellular system by directly inducing expression of anti-ROS genes. Thus, our findings are in line with previous reports that reducing oxidative stress in neurons is beneficial to lifespan.

We further observed that lifespan extension is pronounced in *duox* heterozygous males but less so in heterozygous females, and a similar trend was seen with the neuronal knockdown of the *duox*. Such sexual dimorphism in longevity has previously been observed in other contexts. For example, increased reducing power in neurons via the increased activation of CncC due to *keap1* mutation results in extended lifespan for males, but not females [42]. A recent report similarly found that oltipraz, an NRF2-activating drug, extends lifespan more in males than in females [43]. It appears that sexual dimorphism exists in regard to neuronal oxidative-stress-dependent lifespan.

It is worth mentioning the *duox*-dependent lifespan extension in *C. elegans*. In this model nematode, lifespan is expectably shortened upon the substantive increase of *duox* activity [44]. However, and much more unexpectedly, a modest increase in *duox* results in extended lifespan [44,45]. Why is a low-level increase in *duox* activity beneficial to lifespan? The underlying mechanism involves the activation of the expression of SKN-1, the *C. elegans* homolog of NRF2 [44]. NRF2 is a transcription factor that responds to ROS and is known to extend lifespan [9,45,46,47]. Therefore, extension of lifespan with a low-level increase in *duox* is achieved by strengthening anti-ROS power via NRF2 activation. Thus, our findings concerning *Drosophila duox* in combination with other reports regarding *C. elegans duox* support the notion that oxidative stress regulates lifespan.

Oxidative stress can occur due to an imbalance between ROS production and the cellular antioxidant defense network. In *Drosophila*, NRF2-dependent anti-ROS power declines with age, suggesting that oxidative stress is more likely to occur in older animals [8,43,48,49]. *Duox* activity and expression are increased in gut and embryo epithelial cells undergoing apoptotic cell death, in mammalian neurons through the stabilization of *duox* transcripts by ROS, and in gut epithelial cells by PLCβ-calcium and p38 MAPK signaling [18,24,27,29,30]. In addition, *duox* is elevated in aged brains and in *Drosophila* models of Alzheimer’s disease [50]. Thus, chronic inflammation, protein-aggregate-induced cell death, and various signals involving PLCβ-calcium or p38 MAK could aberrantly activate *duox* activity and expression in neurons, which could confer oxidative stress and lifespan determination. It would be informative to examine whether neuronal *duox* knockdown reduces neuronal inflammation, a hallmark of which is the activation of microglial cells or astrocytes. However, the pursuit of this idea is hindered by the lack of antibodies or markers for activated microglial and astrocytic cells, which is a limitation of these studies.

## Supplementary Materials

The following supporting information can be downloaded at: https://www.mdpi.com/article/10.3390/cells11132059/s1, Figure S1. Negative geotactic movement. Wild-type (*duox*^+/+^) and *duox* heterozygous (*duox*^+/−^) male flies were used for negative geotactic movement. *n* = 100 (10 flies/vial, total 10 vials for each point). *duox*^+/+^ denotes *w^1118^* flies. *duox*^+/−^ denotes *duox ^kG07745/+^* flies, which were isogenized with control (*w^1118^*) flies. Figure S2. Lifespan of female (A–C) and male flies (D). (A) Wild-type and *duox* heterozygous flies. *n* = 400, female flies. *duox*^+/+^ denotes *w^1118^* flies. *duox*^+/−^ denotes *duox ^kG07745/+^* flies, which were isogenized with control (*w^1118^*) flies. (B,C) Lifespan of *elav-Gal4>UAS-duox*^RNAi^ (*elav*/#*38907, 32903*) and *mef-Gal4>* *UAS-duox^RNAi^* (*mef*/#*38907, 32903*). *n* = 200, female flies. (D) Lifespan of *mef-Gal4>* *UAS-duox^RNAi^* (*mef*/#*38907, 32903*). *n* = 200, male flies. *UAS-duox^RNAi^* (*#38907, 32903*) flies were isogenized with control *w^1118^* flies ten times to reduce genetic background differences. Figure S3. Decrease of TUNEL-positive cells in the brain with *duox* heterozygosity and neuronal *duox* reduction. (A,B) Confocal images of whole-mount staining of *Drosophila* (40-day-old) brains with the DNA dye DAPI (blue) and TUNEL (red). (C,D) Quantification of TUNEL-positive cells in panels A & B. Error bars represent SEM of three replicates. Student’s *t*-test, *** *p* < 0.001. *duox*^+/+^ and *duox*^+/−^ denote *w^1118^* and *duox^+/kG07745^*, respectively. *elav*/#*38907, 32903* denotes *elav-Gal4*/ *UAS-duox^RNAi^ #38907, elav-Gal4/+*; *UAS-duox^RNAi^ #32903/+*; Figure S4. Anti-ROS enzyme levels are not affected by *duox* heterozygosity. Agarose gel images of RT-PCR products of anti-ROS genes obtained from male flies. Similar results were obtained among the three replicates. *dSOD* denotes *Drosophila superoxide dismutase*. *rp49* was used as a loading control. ^+/+^ and ^+/−^ denote *w^1118^* and *duox^+/kG07745^*, respectively. Table S1. Quantification of Lifespan.
