# Supplementary material for: Dual Oxidase, a Hydrogen-Peroxide-Producing Enzyme, Regulates Neuronal Oxidative Damage and Animal Lifespan in Drosophila melanogaster
Source: Cells. 2022 Jun 29;11(13):2059. doi: 10.3390/cells11132059 (PMC9265666; doi:10.3390/cells11132059)
Supplement: Supplementary file 1 [file cells-11-02059-s001.zip › cells-1769124-supplementary.pdf]

cells

Supplemental Materials

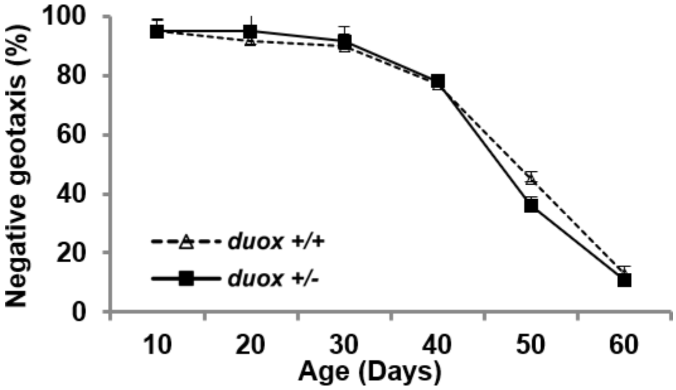

Figure S1. Negative geotactic movement. Wild-type (*duox*$^{+/+}$) and *duox* heterozygous (*duox*$^{+/-}$) male flies wereused for negative geotactic movement. n=100 (10 flies/vial, total 10 vials for each point). *duox*$^{+/+}$ denotes *w*^{1118}flies. *duox*$^{+/-}$ denotes *duox* $^{kG07745/+}$ flies, which were isogenized with control (*w*^{1118}) flies.

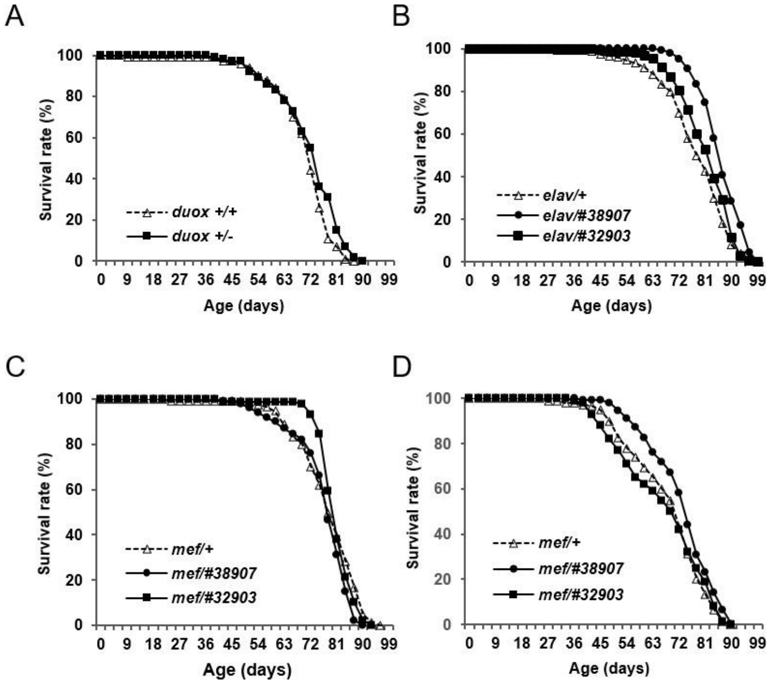

Figure S2. Lifespan of female (**A-C**) and male flies (**D**). (**A**) Wild-type and *duox* heterozygous flies. n=400, female flies. *duox*$^{+/+}$ denotes *w*^{1118} flies. *duox*$^{+/-}$ denotes *duox* $^{kG07745/+}$ flies, which were isogenized with control (*w*^{1118}) flies. (**B,C**) Lifespan of *elav-Gal4>UAS-duox*^{RNAi} (*elav/#38907, 32903*) and *mef-Gal4>UAS-duox* (*mef/#38907, 32903*). n=200, female flies. (**D**) Lifespan of *mef-Gal4>UAS-duox* (*mef/#38907, 32903*). n=200,male flies. *UAS-duox RNAi* (*#38907, 32903*) flies were isogenized with control *w*^{1118} flies ten times to reduce genetic background differences.

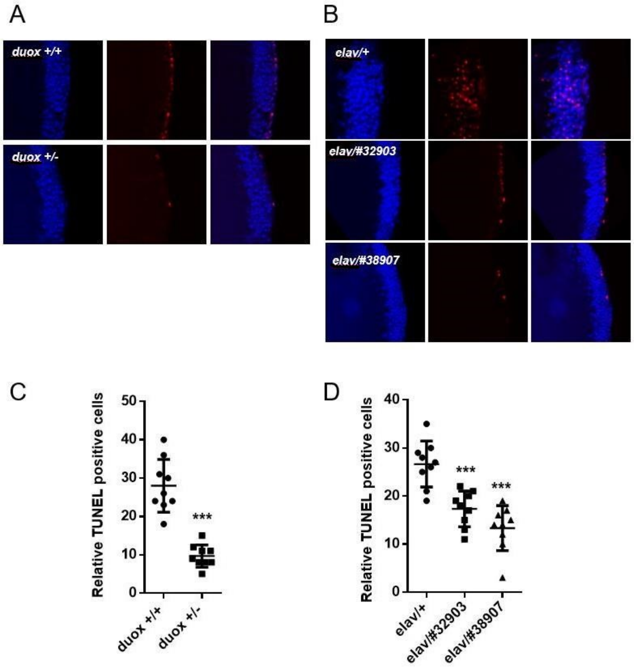

Figure S3. Decrease of TUNEL-positive cells in the brain with *duox* heterozygosity and neuronal *duox* reduction.(**A,B**) Confocal images of whole-mount staining of *Drosophila* (40-day-old) brains with the DNA dye DAPI (blue) and TUNEL (red). (**C,D**) Quantification of TUNEL-positive cells in panels A & B. Error bars represent SEM of three replicates. Student's *t*-test, *** $p < 0.001$. $duox^{+/+}$ and $duox^{+/-}$ denote w^{1118} and $duox^{+/kG07745}$, respectively. *elav/#38907, 32903* denotes *elav-Gal4/UAS-duox RNAi (#38907), elav-Gal4/+;UAS-duox RNAi/+(#32903)*

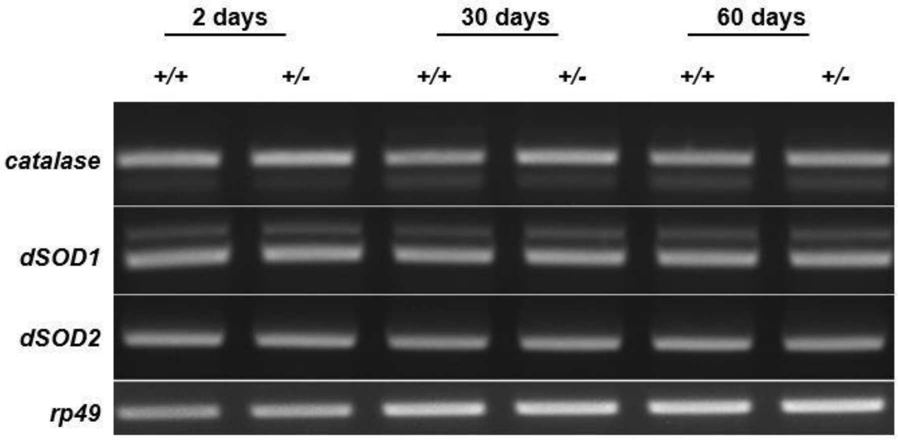

Figure S4. Anti-ROS enzyme levels are not affected by *duox* heterozygosity. Agarose gel images of RT-PCR products of anti-ROS genes obtained from male flies. Similar results were obtained among the three replicates.*dSOD* denotes *Drosophila superoxide dismutase*. *rp49* was used as a loading control. $^{+/+}$ and $^{+/-}$ denote w^{1118} and*duox*$^{+/kG07745}$, respectively.

cells

Table S1. Quantification of Lifespan.

Genotype	Sex	No. of flies	Median lifespan	Change of median lifespan	*p* value
duox +/+	♂	400	70	-	*p* < 0.0001
duox +/-		400	81	15%	
duox +/+	♀	400	70	-	*p* = 0.0156
duox +/-		400	73	4%	

Genotype	Sex	No. of flies	Median lifespan	Change of median lifespan	*p* value
elav/+	♂	200	67	0	
elav/#32903		200	81	20%	*p* < 0.0001
elav/#38907		200	74	10%	*p* < 0.0001
elav/+	♀	200	78	0	
elav/#32903		200	78	5%	*p* = 0.0093
elav/#38907		200	85	9%	*p* < 0.0001
mef/+	♂	200	70	0	
mef/#32903		200	69	0	
mef/#38907		200	73	4%	*p* = 0.0070
mef/+	♀	200	77	0	
mef/#32903		200	79	2%	*p* = 0.2442
mef/#38907		200	77	0	

P-values were obtained by log rank test.
